# A clinical-radiomics nomogram based on dual-layer spectral detector CT to predict cancer stage in pancreatic ductal adenocarcinoma

**DOI:** 10.1186/s40644-024-00700-z

**Published:** 2024-05-09

**Authors:** Linxia Wu, Chunyuan Cen, Xiaofei Yue, Lei Chen, Hongying Wu, Ming Yang, Yuting Lu, Ling Ma, Xin Li, Heshui Wu, Chuansheng Zheng, Ping Han

**Affiliations:** 1 Department of Radiology, Union Hospital, Tongji Medical College, Huazhong University of Science and Technology, 1277 Jiefang Avenue, Wuhan, Hubei Province 430022 The People’s Republic of China; 2 Hubei Province Key Laboratory of Molecular Imaging, Wuhan, 430022 The People’s Republic of China; 3 Regenerative Medicine Research Center, West China Hospital, Sichuan University, Chengdu, Sichuan Province 610041 The People’s Republic of China; 4 Department of Pancreatic Surgery, Union Hospital, Tongji Medical College, Huazhong University of Science and Technology, 1277 Jiefang Avenue, Wuhan, Hubei Province 430022 The People’s Republic of China

**Keywords:** Pancreatic ductal adenocarcinoma, Cancer stage, Dual-layer spectral detector CT, Nomogram, Radiomics

## Abstract

**Background:**

This study aimed to evaluate the efficacy of radiomics signatures derived from polyenergetic images (PEIs) and virtual monoenergetic images (VMIs) obtained through dual-layer spectral detector CT (DLCT). Moreover, it sought to develop a clinical-radiomics nomogram based on DLCT for predicting cancer stage (early stage: stage I-II, advanced stage: stage III-IV) in pancreatic ductal adenocarcinoma (PDAC).

**Methods:**

A total of 173 patients histopathologically diagnosed with PDAC and who underwent contrast-enhanced DLCT were enrolled in this study. Among them, 49 were in the early stage, and 124 were in the advanced stage. Patients were randomly categorized into training (*n* = 122) and test (*n* = 51) cohorts at a 7:3 ratio. Radiomics features were extracted from PEIs and 40-keV VMIs were reconstructed at both arterial and portal venous phases. Radiomics signatures were constructed based on both PEIs and 40-keV VMIs. A radiomics nomogram was developed by integrating the 40-keV VMI-based radiomics signature with selected clinical predictors. The performance of the nomogram was assessed using receiver operating characteristic (ROC) curves, calibration curves, and decision curves analysis (DCA).

**Results:**

The PEI-based radiomics signature demonstrated satisfactory diagnostic efficacy, with the areas under the ROC curves (AUCs) of 0.92 in both the training and test cohorts. The optimal radiomics signature was based on 40-keV VMIs, with AUCs of 0.96 and 0.94 in the training and test cohorts. The nomogram, which integrated a 40-keV VMI-based radiomics signature with two clinical parameters (tumour diameter and normalized iodine density at the portal venous phase), demonstrated promising calibration and discrimination in both the training and test cohorts (0.97 and 0.91, respectively). DCA indicated that the clinical-radiomics nomogram provided the most significant clinical benefit.

**Conclusions:**

The radiomics signature derived from 40-keV VMI and the clinical-radiomics nomogram based on DLCT both exhibited exceptional performance in distinguishing early from advanced stages in PDAC, aiding clinical decision-making for patients with this condition.

**Supplementary Information:**

The online version contains supplementary material available at 10.1186/s40644-024-00700-z.

## Background

Pancreatic cancer remains a highly lethal digestive system disease, with a 5-year survival rate of under 10%. It ranks as the seventh leading cause of cancer-related mortality worldwide [1–3]. Pancreatic ductal adenocarcinoma (PDAC), originating from pancreatic intraepithelial neoplasia, accounts for approximately 90% of pancreatic malignancies [4, 5].

The advanced-stage diagnosis is common in PDAC due to the absence of prominent symptoms in the early stages leading to a delayed diagnosis and treatment initiation [6]. Surgical resection is feasible in early-stage PDAC, offering a five-year survival rate of approximately 20% [7]. However, a majority of patients present with advanced disease, missing the window for surgery [8]. Consequently, primary treatment options involve adjuvant therapies such as radiation and chemotherapy, with a dismal five-year survival rate of approximately 2.9% [6]. Accurate PDAC staging is crucial for assessing disease progression and predicting patient prognosis. However, achieving precise PDAC staging frequently requires invasive operations such as surgical resection and exploratory laparotomy. Endoscopic ultrasound-guided fine-needle aspiration (EUS-FNA) facilitates the acquisition of histological specimens for accurate pathological diagnosis, aiding in determining tumour T staging and peripancreatic lymph node metastasis. However, it entails invasiveness and its accuracy significantly hinges on the operator’s technical proficiency and experience [6]. Therefore, a non-invasive and reliable modality must be developed to differentiate PDAC stages accurately.

While PET-CT and PET-MRI offer advantages in detecting extrapancreatic metastases and assessing overall tumour burden, their high cost diminishes their favourability. Multi-detector computed tomography (MDCT) emerges as the preferred modality for preoperative diagnosis and staging of PDAC due to its cost-effectiveness, and widespread availability [9]. However, the pancreas’ deep-seated location within the abdomen, closely bordered by organs such as the stomach and duodenum, poses challenges for conventional preoperative CT imaging in evaluating occult lesions due to its restricted resolution and assessment parameters [10]. Radiomics has garnered significant attention because it uses advanced image analysis techniques and machine learning algorithms to extract quantitative features from voluminous medical images [11]. In recent years, radiomics has been extensively used in pancreatic tumour diagnosis, preoperative staging, pathological grading, treatment evaluation, and prognosis projection [12–17]. However, these studies primarily rely on conventional CT or MRI images. The advent of spectral CT has brought about a surge in radiomics research rooted in this technology. Dual-layer spectral detector CT (DLCT), a novel form of spectral CT capable of separating low- and high-energy photons, produces more precise images and furnishes more comprehensive energy-related data than conventional CT [18]. Integrating DLCT and radiomics holds significant promise for enhancing the diagnostic and predictive capabilities of radiomic images.

Radiomics utilizing spectral CT demonstrates potential in predicting lymph node metastases in gastric and colorectal cancer, and evaluating gastric cancer response to neoadjuvant therapy [19–21]. However, research on radiomics studies focusing on PDAC rooted in spectral CT remains limited. In this study, our objective was to construct a clinical-radiomics nomogram based on DLCT, providing a non-invasive method to differentiate early- and advanced-stage PDAC.

## Methods

### Participants

**Fig. 1:**
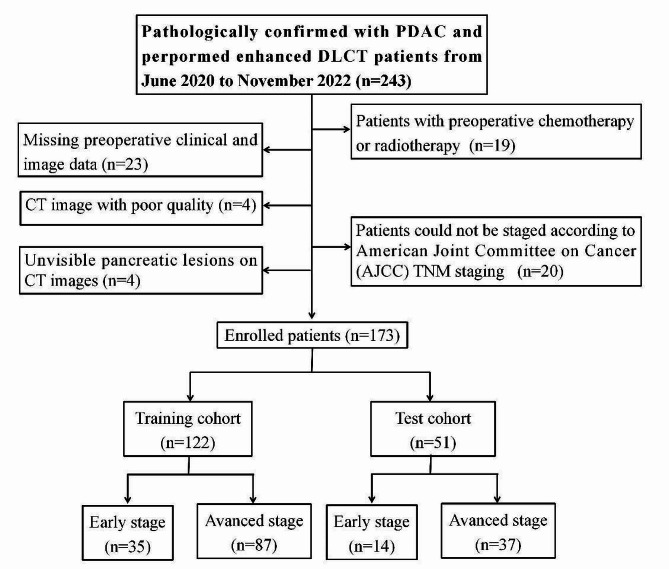
Flow chart of the patient selection process

Ethical approval was obtained from the ethical committee of Tongji Medical College, Huazhong University of Science and Technology, following the Declaration of Helsinki. Due to the retrospective design, informed consent was waived. Between June 2020 and November 2022, 173 consecutive patients with pathologically confirmed PDAC were enrolled in this study. The inclusion criteria were as follows: (a) pathologically confirmed PDAC diagnosis; (b) ability to stage patients based on Union for American Joint Committee on Cancer (AJCC) Tumour-Nodal involvement-Metastasis (TNM) staging (eighth edition) [10]; and (c) patients with PDAC who underwent preoperative DLCT scanning within 2 weeks. The exclusion criteria were as follows: (a) preoperative chemotherapy or radiotherapy; (b) inadequate CT quality; (c) pancreatic cancer lesions too small for accurate evaluation; and (d) incomplete clinical information. The flow diagram illustrating PDAC patient enrolment is depicted in Fig. 1. Clinical data, including age, gender, comorbidities, tumour diameter, tumour number, tumour location, levels of carbohydrate antigen 19 − 9 (CA19-9), carbohydrate antigen 12 − 5 (CA12-5), and carcinoembryonic antigen (CEA), were collected from electronic medical records. The training group was utilized to construct the model and fine-tune parameters during cross-validation. In contrast, the test group evaluated the model’s generalization performance without involvement in feature selection, feature standardization, and model construction.

### CT image acquisition

Details of the DECT scan technique are provided in Supplementary Material 1. Polyenergetic images (PEIs) were generated using an iterative reconstruction algorithm (iDose 4, level 3; Philips Healthcare) to represent conventional CT image sets and then transferred to the picture archiving and communication system. Furthermore, 40-keV virtual monoenergetic images (VMIs) [22–24] and other spectral-based imaging datasets, including material-decomposition maps of iodine density (ID) and effective atomic numbers (Z_eff_) maps, were generated using the postprocessing workstation (IntelliSpace Portal 9.0, Philips Healthcare).

### CT spectral parameters measurement

In the Philips ISP postprocessing workstation, spectral parameters were assessed by placing a region of interest (ROI) within the tumour lesion. Two abdominal radiologists, each with 5 and 7 years of post-training experience (L.W. and C.C.), independently reviewed the DLCT examinations for each patient. Axial 40-keV VMI images at both the arterial and portal venous phases were chosen, and a manually delineated ROI was positioned within the tumour lesion, following these guidelines: (1) The ROI was positioned to exclude necrotic areas, large blood vessels, and dilated pancreatic ducts. (2) The ROI area should have exceeded two-thirds of the maximum area of the tumour lesion on the axial image. (3) The ROI’s shape, size, and position for the lesion within the same patient should have remained consistent between the 40-keV VMI and PEI. For each tumour, three ROIs were positioned: at the lesion’s superior margin, the level of its maximum axial diameter, and its inferior margin. Subsequently, the delineated ROI was replicated on other spectral images to measure the CT values, Z_eff_, and ID values.

The mean values of the three ROIs were recorded to obtain overall measurements for each lesion. ID values were measured in the abdominal aorta at the same position as the lesions during the arterial and portal venous phases. Subsequently, the normalized ID (NID) was calculated using Formula (1) [21] as the ratios of these values between the tumour and the abdominal aorta during the specific phase. The slope of the attenuation curve (K-slope) was determined by utilizing the arterial and portal venous phase VMIs within the energy range of 40-keV to 100-keV, as demonstrated in Formula (2) [25]. Interobserver agreement was assessed for the quantitative spectral parameters (Supplementary Table 1). The mean values of the measurements from both readers were then used for all subsequent analyses.

1 $${\rm{NID}}\,{\rm{ = }}\,{{{\rm{tumor}}\,{\rm{lesions}}\,{\rm{ID}}} \over {{\rm{same}}\,{\rm{level}}\,{\rm{aorta}}\,{\rm{ID}}}}$$

2 $${\rm{K}}\, - \,{\rm{slope}}\,{\rm{ = }}\,{{HU{\,_{{\rm{40}}keV}}\, - \,H{U_{{\rm{100}}keV}}} \over {{\rm{100}}\, - \,{\rm{40}}}}$$

### Final diagnosis of TNM stage

The TNM staging for all patients was assessed following the guidelines outlined in the eighth edition of the AJCC TNM staging. According to this criterion and studies focusing on the survival outcomes of patients PDAC patients at various stages [10, 26], the mortality rates associated with stage I or II disease were lower than those correlated with stage III or IV disease. Therefore, the TNM staging of PDAC was aimed to be simplified into early-stage (stages I and II) and advanced-stage (stages III and IV) disease.

The final diagnoses of TNM stage and each category were reached by consensus at the multi-disciplinary treatment (MDT) board conference attended by diagnostic radiologists, pathologists, and pancreatic surgeons with extensive experience ranging from 17 to 26 years.

### Tumour segmentation, image preprocessing, and radiomics feature extraction

The workflow of the radiomics analysis is illustrated in Supplementary Fig. 1.

The three-dimensional (3D) ROI of the tumour was manually delineated slice-by-slice on arterial phase (AP) and portal venous phase (PVP) CT images (40-keV VMI and PEI) using ITK-SNAP software (www.itksnap.org). The ROIs of all patients with PDAC were segmented by two radiologists, each with over 5 years of experience in abdominal imaging diagnosis; both were blinded to the corresponding pathological results.

Despite originating from DLCT, all images underwent preprocessing to enhance the model’s generalization performance. Before feature extraction, images were resampled with a voxel size of 1 × 1 × 1 mm³. Moreover, all CT images were normalized to a scale of 500. Grey-level discretization was performed on the original intensities and resampled to 25 bins with a fixed number of bins (more details in Supplementary Material 1).

Subsequently, using the Python package “PyRadiomics” in Anaconda Prompt software (version 4.2.0) (github.com/Radiomics/PyRadiomics), radiomics features were extracted for each 3D ROI. Both intraobserver and interobserver analyses were conducted to evaluate the reproducibility of radiomics features. This assessment was accomplished using the intra- and interclass correlation coefficients (ICCs). Specifically, these coefficients were employed to gauge the agreement between the features generated by X.Y. (first time) and those generated by L.W. Furthermore, the agreement between features generated twice by L.W. was also evaluated. ICCs were classified as: <0.50, poor agreement; 0.50–0.75, moderate agreement; 0.75–0.90, good agreement; and > 0.90, excellent agreement [27].

### Feature selection and radiomics signature construction

All feature selection processes were conducted in the training dataset. Maximum relevance minimum redundancy (mRMR) methods and the Least absolute shrinkage and selection operator (LASSO) logistic regression were applied to the training cohort to identify the most stable and predictive features. Features with nonzero coefficients were selected to construct the radiomics signature. A linear combination of these features and their corresponding weight coefficients established the radiomics signature, and the radiomics score (Radscore) for each patient was computed.

### Clinical model and clinical-radiomics model construction

Clinical model construction: Univariate and stepwise regression analyses were applied, and based on the principle of minimizing the Akaike information criterion (AIC), essential clinical factors associated with the PDAC stage were identified to establish a clinical model.

Construction of the clinical-radiomics model construction: Multivariable logistic regression analysis was conducted to ascertain independent predictors, and the radiomics signature (Radscore) was considered an independent risk factor. It was combined with crucial clinical features to construct a clinical-radiomics model. Variance inflation factors of the predictors were computed to diagnose collinearity.

### Model validation and evaluation

The constructed radiomics, clinical, and clinical-radiomics models were evaluated in terms of diagnostic performance, goodness of fit, and clinical utility. A receiver operating characteristic (ROC) curve was employed to assess the discriminative ability of the three models, and the area under the ROC curve was calculated separately in the training and test cohorts. Calibration curves were plotted to determine the agreement between prediction and observation in the three models. Clinical decision curve analysis (DCA) was used to determine the net benefit rate of the three models.

### Statistical analysis

All statistical analyses were conducted with R software, version 4.2.0 (The R Foundation for Statistical Computing; http://www.r-project.org). Quantitative data were presented as mean ± standard deviation (X ± SD) or median and quartiles [M(P25, P75)]. Qualitative data are expressed as percentages (%). Comparisons between the two groups were performed using the t-test or the Mann-Whitney U test for quantitative variables, and the χ2 or Fisher’s test for qualitative variables. All statistical tests were two-sided, and statistical significance was set at *p* < 0.05.

The R packages used in this study comprised “tidyverse”, “caret”, “pROC”, “glmnet”, “rmda”, “gpub”, “ModelGood”, “DMwR2”, “mRMRe”, “DescTools”, “mRMRe”, “DescTools” and “Publish”.

## Results

### Patient demographics

Among the 173 patients enrolled in this study, 106 were men and 67 were women, with an average age of 61 ± 9 years. According to the AJCC eighth edition pancreatic cancer staging criteria, the patients with PDAC were classified as early-stage (49 cases, stages I: 20 and stage II: 29) and advanced-stage (124 cases, stages III: 72 and stage IV: 52) groups. The patient cohort was further divided into a training (*n* = 122) and a test cohort (*n* = 51) at a ratio of 7:3. The baseline characteristics of patients in the training and test cohorts are summarized in Supplementary Table 2, with no significant differences observed in the baseline data between the training and test cohorts (*p* > 0.05). Table 1 illustrates the variations in clinical features and CT spectral parameters between patients at early and advanced stages within the training and test cohorts.

**Table 1:** Baseline characteristics of the PDAC stage model

Characteristics	Training cohort (*n* = 122)		*p* value	Test cohort (*n* = 51)		*p* value
	Early stage (*n* = 35)	Advanced stage (*n* = 87)		Early stage (*n* = 14)	Advanced stage (*n* = 37)	
Age (y)	60.9 ± 6.1	61.9 ± 9.4	0.570	63.2 ± 5.6	58.3 ± 10.8	0.107
Gender			0.380			0.912
Male	18 (51.4)	54 (62.1)		10 (71.4)	24 (64.9)	
Female	17 (48.6)	33 (37.9)		4 (28.6)	13 (35.1)	
BMI	22.5 ± 3.3	22.0 ± 3.1	0.420	23.2 ± 2.5	22.2 ± 3.6	0.322
Smoking	7 (20.0)	20 (23.0)	0.906	3 (21.4)	6 (16.2)	0.981
Diabetes	11 (31.4)	15 (17.2)	0.137	1 (7.1)	5 (13.5)	0.886
Tumour location			0.037*			0.052
Head and neck	26 (74.3)	45 (51.7)		11 (78.6)	16 (43.2)	
Body and tail	9 (25.7)	42 (48.3)		3 (21.4)	21 (56.8)	
Tumour diameter			< 0.001*			0.004*
≤ 2 cm	8 (22.9)	4 (4.6)		1 (7.1)	1 (2.7)	
> 2–4 cm	24 (68.6)	36 (41.4)		12 (85.7)	14 (37.8)	
> 4 cm	3 (8.5)	47 (54.0)		1 (7.2)	22 (59.5)	
CA19-9			0.644			0.244
< 37 U/ml	8 (22.9)	15 (17.2)		6 (42.9)	8 (21.6)	
≥ 37 U/ml	27 (77.1)	72 (82.8)		8 (57.1)	29 (78.4)	
CA12-5			0.002*			0.937
< 35 U/ml	27 (77.1)	39 (44.8)		10 (71.4)	26 (70.3)	
≥ 35 U/ml	8 (22.9)	48 (55.2)		4 (28.6)	11 (29.7)	
CEA			0.086			0.608
< 5 ug/L	22 (62.9)	38 (43.7)		9 (64.3)	19 (51.4)	
≥ 5 ug/L	13 (37.1)	49 (56.3)		5 (35.7)	18 (48.6)	
CT_Hu-_AP (Hu)	107.0 ± 36.5	76.0 ± 23.1	< 0.001*	102.5 ± 40.8	78.7 ± 24.5	0.011*
CT_Hu-_PVP (Hu)	145.9 ± 44.5	105.4 ± 24.4	< 0.001*	138.4 ± 56.0	105.3 ± 26.8	0.004*
ID-AP (mg/ml)	0.8 ± 0.5	0.5 ± 0.3	< 0.001*	0.8 ± 0.4	0.5 ± 0.3	0.010*
ID-PVP (mg/ml)	1.4 ± 0.6	0.9 ± 0.3	< 0.001*	1.2 ± 0.6	0.9 ± 0.3	0.006*
NID-AP	0.09 ± 0.05	0.05 ± 0.03	< 0.001*	0.07 ± 0.03	0.05 ± 0.03	0.028*
NID-PVP	0.3 ± 0.1	0.2 ± 0.1	< 0.001*	0.3 ± 0.1	0.2 ± 0.1	0.010*
IDD (mg/ml)	1.0 ± 0.6	0.8 ± 0.5	0.014*	0.9 ± 0.8	0.7 ± 0.4	0.218*
K-slope-AP	1.1 ± 0.6	0.6 ± 0.3	< 0.001*	1.0 ± 0.6	0.7 ± 0.4	0.006*
K-slope-PVP	1.6 ± 0.6	1.1 ± 0.4	< 0.001*	1.6 ± 0.9	1.1 ± 0.4	0.005*
Z_eff_-AP	7.8 ± 0.3	7.6 ± 0.2	< 0.001*	7.8 ± 0.3	7.6 ± 0.2	0.008*
Z_eff_-PVP	8.1 ± 0.3	7.8 ± 0.2	< 0.001*	8.0 ± 0.4	7.8 ± 0.2	0.011*

*Note* Data are n (%) or mean (standard deviation); data in parentheses are percentages

*Abbreviation* AP, arterial phase; PVP, portal venous phase; ID, iodine density; NID, normalized iodine density; IDD, Iodine density difference (ID-AP-ID-PVP); CT, computed tomography; CA 19–9, carbohydrate antigen 19–9; CA 12–5, carbohydrate antigen 12–9; CEA, carcinoembryonic antigen. K-slope, slope of the attenuation curve; Z_eff_, effective atomic number

*p value < 0.05

### Extraction of radiomics features and calculation of Radscore

**Fig. 2:**
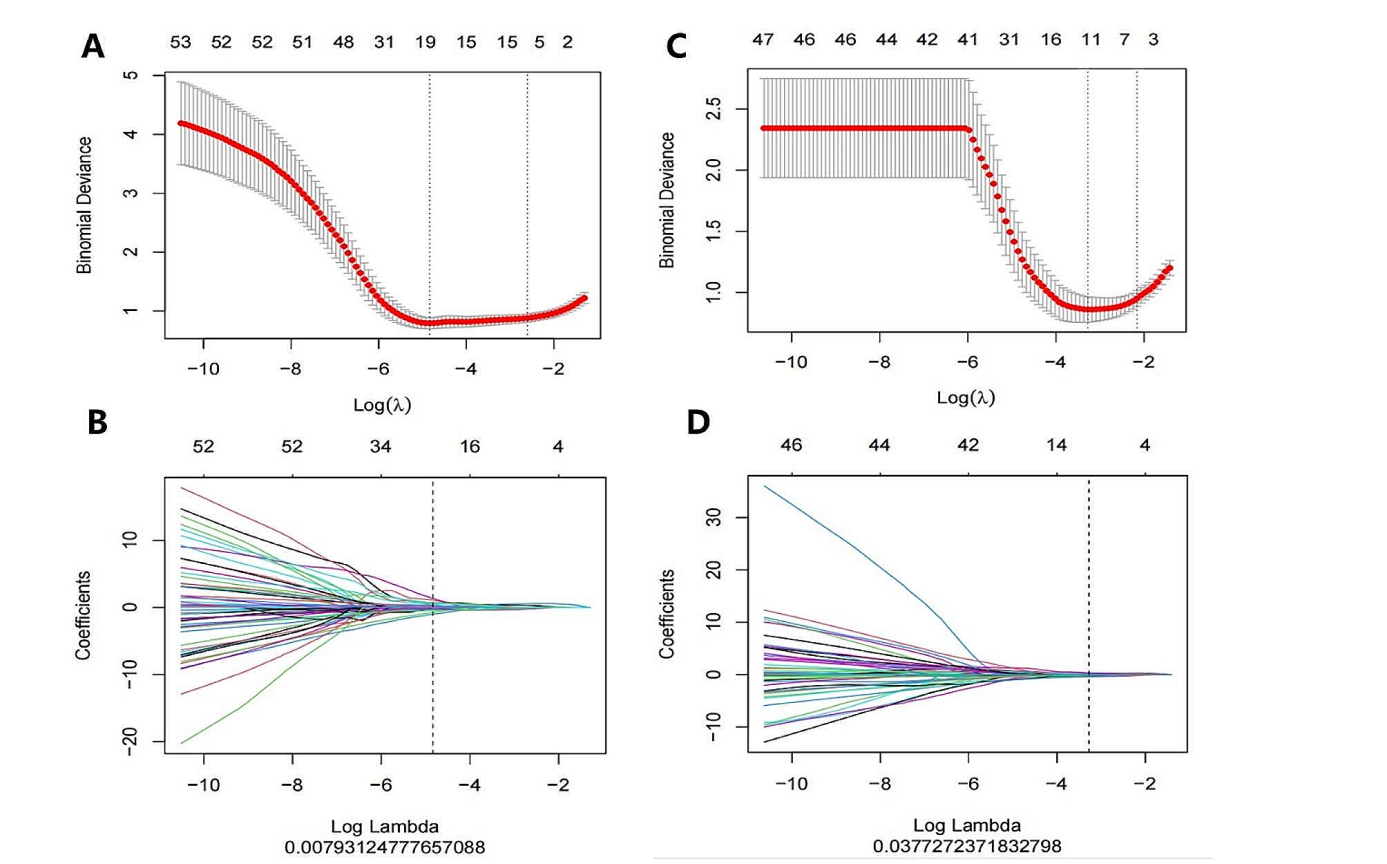
Radiomics features selection with the LASSO logistic regression model. (**A**). 40-keV VMI-based and (**B**). PEI-based radiomics features selection used LASSO with 10-fold cross-validation by the minimum criteria. (**C**). 40-keV VMI-based and (**D**). PEI-based radiomics features of the LASSO coefficient profiles. Y-axis indicates binomial deviances. The upper x-axis indicates the average number of radiomics features. The lower x-axis indicates the log(λ) value

A total of 1,218 features were extracted from the ROI based on arterial and portal venous CT images. Detailed radiomics features are provided in Supplementary Fig. 2. A random selection of 30 patients was made for intraobserver analysis, resulting in ICCs of 0.94 ± 0.10 and 0.92 ± 0.10 for 40-keV VMI-based and PEI-based radiomics features, respectively. For interobserver analysis, the results were 0.86 ± 0.15 and 0.85 ± 0.19, respectively. Features with intra- and interobserver ICCs > 0.75 (indicating good stability) were selected. Subsequently, the mRMR algorithm and LASSO regression were employed for further feature selection (Fig. 2). Ultimately, 19 optimal radiomics features were chosen to construct the 40-keV VMI-based radiomics model. In comparison, 11 optimal radiomics features were selected for the PEI-based model. The selected features and their corresponding coefficients are presented in Supplementary Fig. 3. Equations for the two radiomics models are presented in Supplementary Material 2. The Radscore for the 40-keV VMI-based and PEI-based models were calculated separately utilizing the screened features and the corresponding coefficients. To differentiate between the two Radscores, the one derived from the 40-keV VMI model was designed as Radscore_40keV_ and the one from the PEI model as Radscore_PEI_.

### Performance of the Radscore

**Fig. 3:**
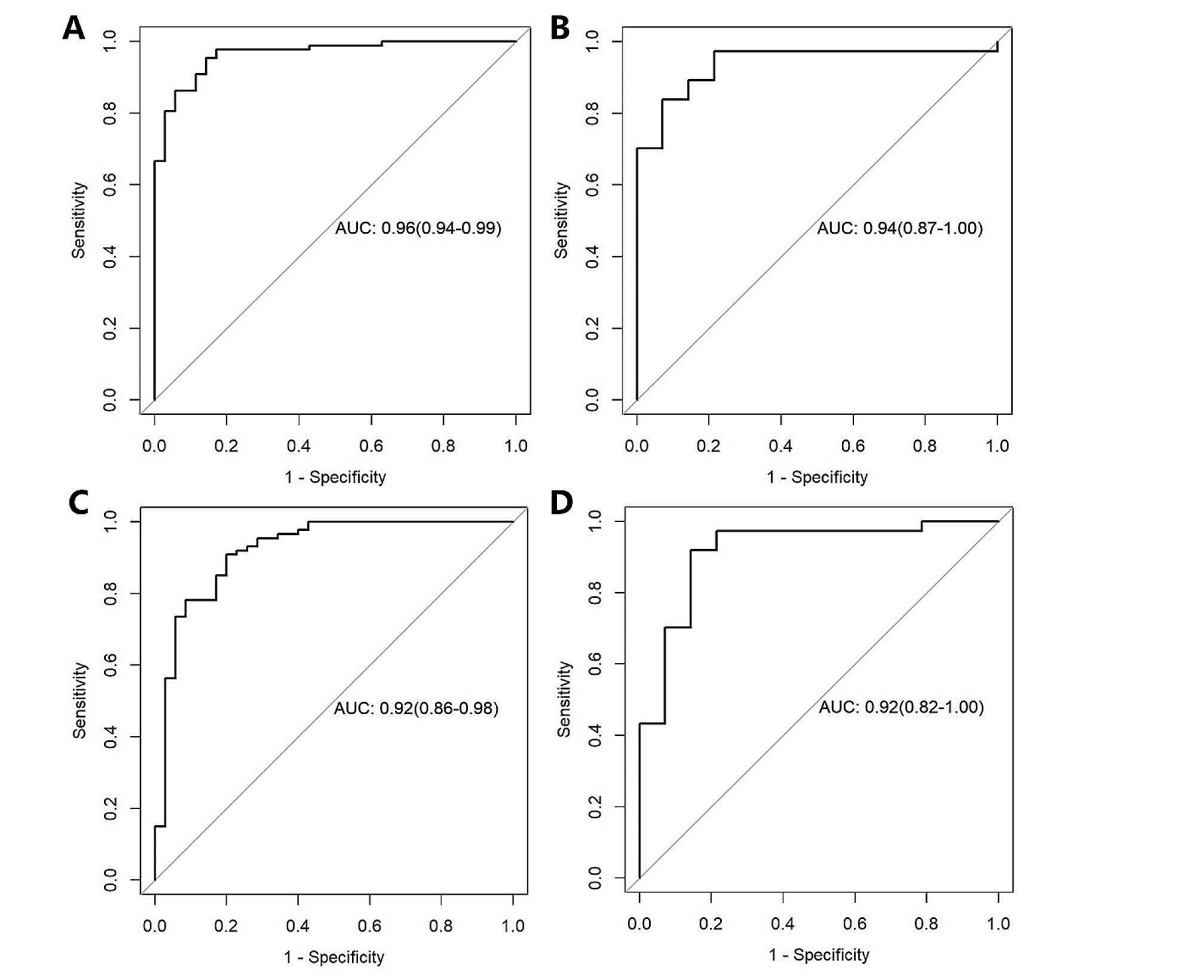
The performance of the radiomics signature for preoperative diagnosis of PDAC stage. ROC curve of the 40-keV VMI-based radiomics signature in (**A**). the training cohort and (**B**). the test cohort. ROC curve of the PEI-based radiomics signature in (**C**). the training cohort and (**D**). the test cohort

Using the optimal cutoff values (Radscore40_keV_, − 0.008; Radscore_PEI_, 0.651) determined from the training cohort based on the maximum Youden index, the test cohort underwent dichotomous classification. Substantial differences in Radscore_40keV_ and Radscore_PEI_ were observed between early-stage and advanced-stage patients with PDAC in both the training and test cohorts (*p* < 0.001) (Supplementary Fig. 4). Radscore_40keV_ exhibited a favourable AUC of 0.96 (95% CI, 0.87–0.96) in the training cohort and 0.94 (95% CI, 0.87-1.00) in the test cohort (Fig. 3A and B). Radscore_PEI_ yielded an AUC of 0.92 (95% CI, 0.86–0.98) in the training cohort and 0.92 (95% CI, 0.82–1.00) in the test cohort (Fig. 3C and D). The sensitivity (training cohort: 0.95; test cohort: 0.97) and accuracy (training cohort: 0.93; test cohort: 0.92) of Radscore_40keV_ surpassed those of Radscore_PEI_ (sensitivity: training cohort: 0.91, test cohort: 0.86; accuracy: training cohort: 0.88, test cohort: 0.86) (Table 2). While both models demonstrated good diagnostic performance, Radscore_40keV_ outperformed Radscore_PEI_.

**Table 2:** The performance of Radscore_40keV_and Radscore_PEI_

	Radscore_40keV_		Radscore_PEI_	
	Training cohort	Test cohort	Training cohort	Test cohort
AUC	**0.96 (0.94–0.99)**	0.94 (0.87-1.00)	0.92 (0.86–0.98)	0.92 (0.82-1.00)
ACC	**0.93(0.86–0.97)**	0.92(0.81–0.98)	0.88 (0.81–0.93)	0.86(0.74–0.94)
SENS	**0.95 (0.89–0.99)**	0.97 (0.86–0.99)	0.91 (0.83–0.96)	0.86 (0.71–0.96)
SPEC	**0.86 (0.70–0.95)**	0.79 (0.49–0.95)	0.80 (0.63–0.92)	0.86 (0.57–0.98)
PPV	**0.94 (0.88–0.97)**	0.92 (0.82–0.97)	0.92 (0.85–0.96)	0.94 (0.82–0.98)
NPV	0.88 (0.74–0.95)	**0.92 (0.61–0.99)**	0.78 (0.64–0.87)	0.71 (0.51–0.85)

*Note* The numbers in parentheses are the 95% confidence interval. The bold values represent the optimal value

*Abbreviation* AUC, areas under receiver operating characteristic curve; ACC, accuracy; SENS, sensitivity; SPEC, specificity; PPV, positive predictive value; NPV, negative predictive value

### Clinical-radiomics model

Utilizing the minimum AIC principle (AIC min = 102.42), stepwise regression analysis identified independent clinical risk factors influencing PDAC staging. Finally, two clinical features, namely, tumour diameter and NID at the portal venous phase (NID-PVP), were selected to construct the clinical model. The variance inflation factors (VIFs) of the two clinical variables were less than five, indicating no collinearity.

**Fig. 4:**
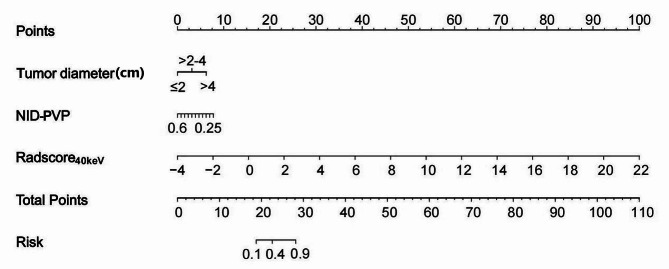
Development of the 40-keV VMI-based radiomics nomogram and its performance. Mapping a line upwards based on the actual values of each variable onto the ***Points*** scale is performed to determine the score for each variable. The scores for all variables are then summed, and a line is drawn downwards to establish the overall score on the column chart, denoted as the ***Total Points***. This process is used to predict the ***Risk*** probability of a patient having advanced-stage pancreatic cancer

Compared with the PEI-based radiomics model, the 40-keV VMI-based radiomics model demonstrated potentially superior performance. Therefore, the 40-keV VMI-based radiomics model was chosen to construct the clinical-radiomics model. An integrated diagnostic model was developed by incorporating clinical variables using logistic regression. Tumour diameter and NID-PVP emerged as independent factors in the clinical-radiomics model through multivariate logistic regression. The VIFs for the three factors were 1.477, 1.396 and 1.691, respectively. The nomogram incorporating clinical factors and Radscore_40keV_ is shown in Fig. 4.

### Model evaluation

**Fig. 5:**
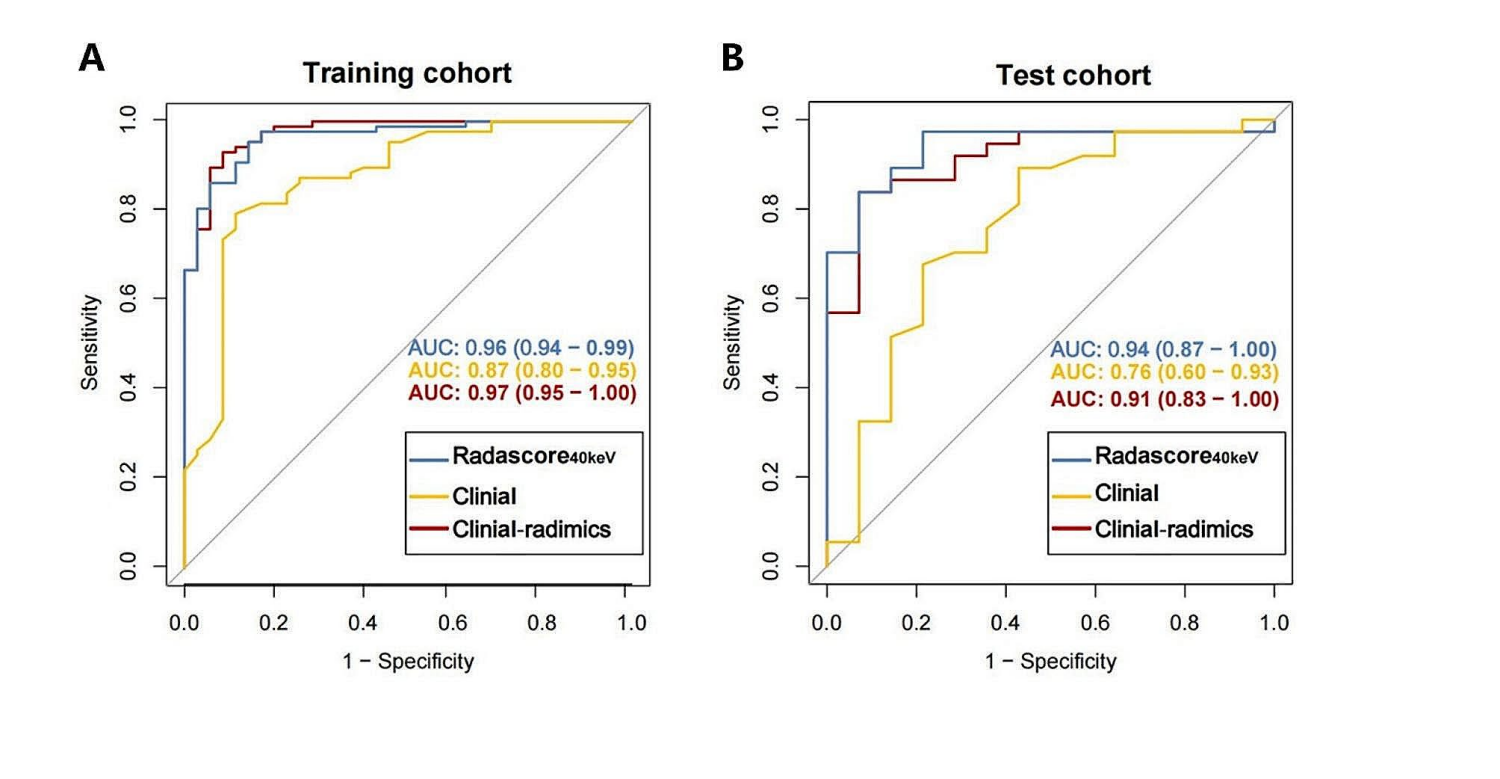
Diagnostic performance of clinical-radiomics model for differentiation of early and advanced stage PDAC. The ROC curves of Radscore_40keV_, clinical model and clinical-radiomics model in (**A**) the training cohort and (**B**). the test cohort. The red line represents the clinical-radiomics model, the blue line represents the Radscore_40keV_ and the yellow line represents the clinical model

Optimal cutoff values of 1.175 and 0.625 were selected as the stratification thresholds for the clinical model and clinical-radiomics model, respectively. Significant differences were observed between the early- and advanced-stage groups of the clinical-radiomics model in both the training and test cohorts (*p* < 0.001). ROC analysis indicated that the AUC of the clinical-radiomics model in the training cohort (Fig. 5A) was 0.97 (95% CI: 0.95–1.00), and in the test cohort (Fig. 5B), it was 0.91 (95% CI: 0.83–1.00). In contrast, the AUC of the clinical model in the training cohort was 0.87 (95% CI: 0.80–0.95), and in the test cohort was 0.76 (95% CI: 0.60–0.93). The sensitivity, specificity, and accuracy of the three models are detailed in Table 3.

**Table 3:** The performance of different models based on 40 keV VMI

Models	AUC	SENS	SPEC	ACC
Radiomics model				
Training cohort	0.96 (0.94–0.99)	0.95 (0.89–0.99)	0.86 (0.70–0.95)	0.93 (0.86–0.97)
Test cohort	0.94 (0.87-1.00)	**0.97 (0.86–0.99)**	0.79 (0.49–0.95)	0.92 (0.81–0.98)
Clinical model				
Training cohort	0.87 (0.80–0.95)	0.79 (0.69–0.87)	0.89 (0.73–0.97)	0.82 (0.74–0.88)
Test cohort	0.76 (0.60–0.93)	0.70 (0.53–0.84)	0.71 (0.42–0.92)	0.71 (0.56–0.83)
Clinical-radiomics model				
Training cohort	**0.97 (0.95-1.00)**	0.93 (0.86–0.97)	**0.91 (0.77–0.98)**	**0.93 (0.86–0.98)**
Test cohort	0.91 (0.83-1.00)	0.89 (0.75–0.97)	0.71 (0.42–0.92)	0.84 (0.71–0.93)

*Note* The numbers in parentheses are the 95% confidence interval. The bold values represent the optimal value

*Abbreviation* VMI, virtual monoenergetic image; AUC, areas under receiver operating characteristic curve; ACC, accuracy; SENS, sensitivity; SPEC, specificity

Calibration curves exhibited satisfactory calibration capacity for all three models in both the training and test cohorts (Supplementary Fig. 5). Results from the Hosmer‒Lemeshow test indicated no significant differences between the diagnoses from the radiomics model (*p* = 0.905), clinical model (*p* = 0.606), and clinical-radiomics model (*p* = 0.741) in the training cohort, or between the radiomic model (*p* = 0.154), clinical model (*p* = 0.187), and clinical-radiomics model (*p* = 0.513) in the test cohort when compared with the actual results.

**Fig. 6:**
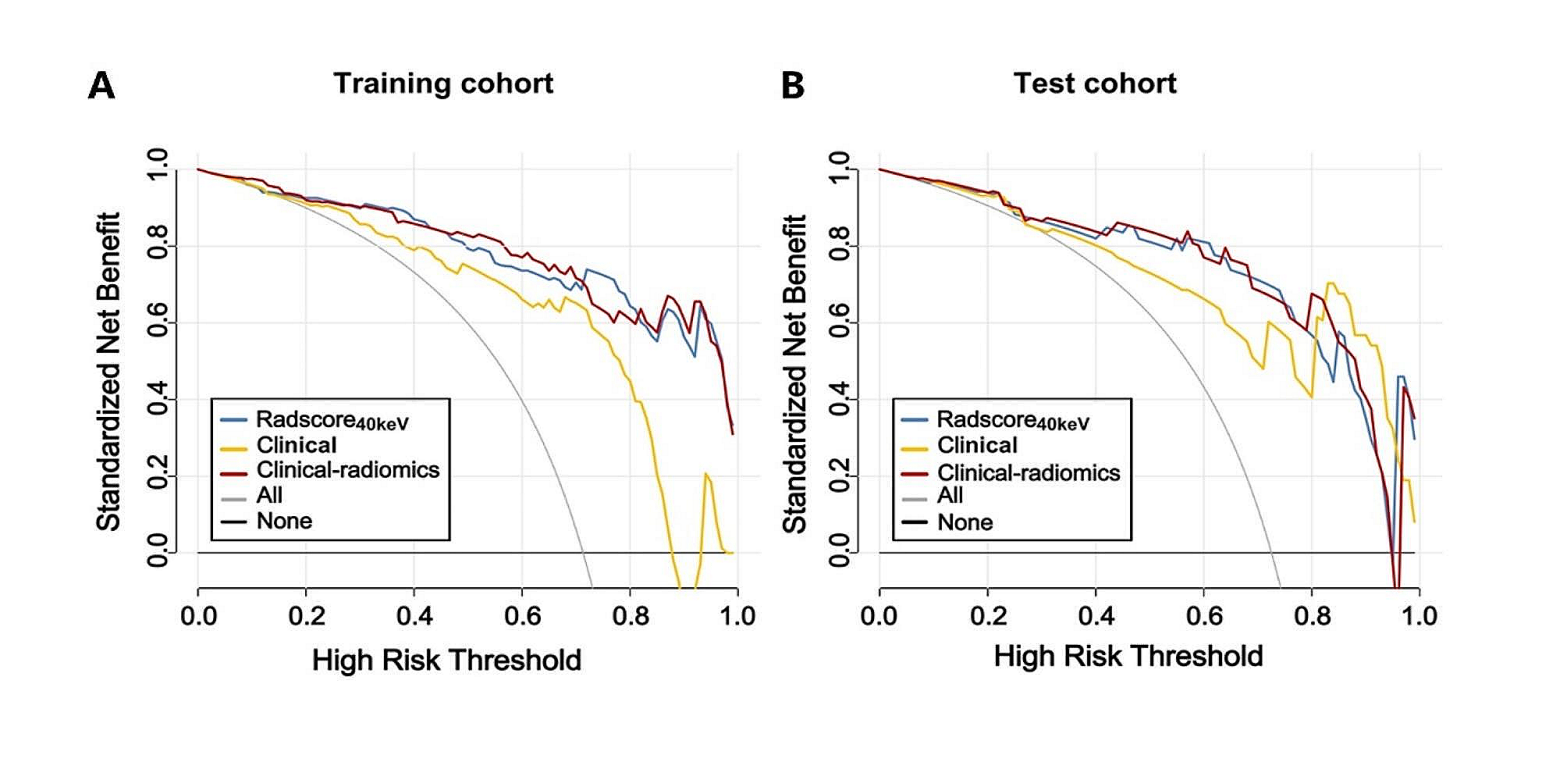
Decision curves for Radscore_40keV_, clinical model and clinical-radiomics model in (**A**). the training cohort and (**B**). the test cohort. The red line represents the clinical-radiomics model. The blue line represents the Radscore_40keV_ and the yellow line represents the clinical model. The X-axis means the threshold probability, Y-axis shows the model benefit

Decision curves revealed that the net benefit corresponding to the clinical-radiomics and radiomics models significantly exceeded that of the clinical model at different threshold probabilities (Fig. 6).

## Discussion

This study aimed to develop a non-invasive clinical-radiomics model based on DLCT for preoperative prediction of the PDAC stage. The spectral DLCT-based nomogram integrated a 19-feature radiomics signature and clinical factors, including tumour diameter and NID-PVP. Our findings demonstrated that the clinical-radiomics nomogram demonstrated favourable performance and might be an effective tool for PDAC staging and clinical decision-making.

Spectral CT allows simultaneous acquisition of images using two different reconstruction methods, the mixed energy image (PEI) generated by iDose4 iterative reconstruction technology and the single-energy image (VMI) obtained from the spectral-based imaging data packet (SBI). Low-energy level VMI from DLCT provides superior image quality, particularly for PDAC evaluation. Specifically, the 40-keV VMI offers the most precise depiction of tumour lesions and surrounding pancreatic vessels without an increase in image noise [22–24]. Therefore, the utility of a 40-keV VMI-based radiomics model was investigated to assess the PDAC stage, demonstrating promising diagnostic performance. Furthermore, a PEI-based radiomics model was constructed and compared with the 40-keV VMI-based radiomics model. Our results revealed that the diagnostic performance of the 40-keV VMI-based radiomics model (training cohort AUC = 0.96; test cohort AUC = 0.94) surpassed that of the PEI-based radiomics model (training cohort AUC = 0.92; test cohort AUC = 0.92). The sensitivity (0.95, 0.97) and accuracy (0.93, 0.92) of the former were superior to those of the latter (sensitivity: 0.91, 0.86; accuracy: 0.88, 0.86) in both the training and test cohorts.

A previous DLCT imaging study for PDAC highlighted significantly lower image noise in VMI than in PEI. Moreover, VMI’s contrast-to-noise ratios (CNRs) exhibited a notable increase relative to PEI, with CNRs progressively rising with energy decrease. Subjective visual assessment scores peaked at 40-keV VMI, followed by 50-keV and 60-keV VMIs, all significantly outperforming PEI [24]. Furthermore, other studies have corroborated that VMI at 40 keV offers superior lesion detectability compared to conventional PEI [28, 29]. These findings provide a theoretical basis for the exceptional performance of the 40-keV VMI-based model in this study compared to the PEI-based model.

An increasing number of model development approaches are moving beyond pure radiomics model studies. Instead, they incorporate clinical features alongside radiology to construct combined clinical-radiomics models, enhancing diagnostic or predictive performance [30–32]. Hence, this study employed multivariable logistic regression to identify the most valuable clinical factors for PDAC staging. By integrating clinical factors with the radiomics signature, a comprehensive model with higher predictive efficacy was constructed. The pivotal factors selected in this study were tumour diameter and NID-PVP. This selection was consistent with previous research; for instance, Cen et al. identified tumour diameter as a crucial clinical factor for PDAC staging, reaffirming its significance [33]. Moreover, various studies have found that iodine density values (ID), especially NID, hold substantial value in the early diagnosis of pancreatic cancer, differential diagnosis with mass-forming chronic pancreatitis, evaluation after chemoradiotherapy, recognition of lymph node metastasis, and prediction of postoperative recurrence [34–38]. Given the superior performance of the 40-keV VMI-based radiomics model, it was deemed an independent predictive factor. It was combined with NID-PVP and tumour diameter to develop a comprehensive clinical-radiomics model, to enhance diagnostic accuracy. The results demonstrated satisfactory performance with an AUC of 0.97 in the training cohort and 0.91 in the test cohort, surpassing those of the pure clinical model (training cohort: 0.87, training cohort: 0.76). Previous studies integrated tumour location, tumour size, CEA, and radiomics features to construct a clinical-radiomics model for PDAC staging, achieving AUCs of 0.92 in the training cohort and 0.83 in the test cohort [33]. Our findings outperformed these results, possibly attributed to the marginally inferior display of PDAC lesions in conventional CT images compared to that of spectral CT mono-energy images, which affects accurate identification and delineation [39]. In contrast, the 40-keV VMI offers a more precise visualization of PDAC lesions and surrounding vessels, facilitating accurate ROI delineation and improving the accuracy of lesion segmentation. NID-PVP enables quantitative analysis of lesion enhancement levels, which is more precise and objective than traditional visual observation.

Another vital aspect to consider in clinical prediction models is their clinical utility. Calibration curves and DCA were employed to assess the performance of the DLCT-based clinical-radiomics nomogram [40]. The results indicate that the clinical-radiomics nomogram outperforms the pure clinical model in the training and test cohorts. Moreover, the two predictive indicators integrated into the combined nomogram are readily obtainable from routine examinations. Therefore, the CT-based nomogram can be a non-invasive, convenient, and accessible tool for preoperative differentiation between early- and advanced-stage PDAC.

In current clinical practice, aside from TNM (AJCC) staging, the preoperative staging system commonly used to aid clinicians in decision-making includes resectability assessment. The most widely-used National Comprehensive Cancer Network (NCCN) guideline classifies PDAC resectability into three groups based on the degree of tumour vascular contact: resectable, borderline resectable, and locally advanced disease, as determined by preoperative enhanced CT [9]. However, assessment based on CT of significant vascular invasion is highly subjective and dependent on experienced radiologists [41]. A prospective multicentre study reported significant interobserver variability, with an agreement of only 0.55 in determining vascular involvement by CT [42]. Furthermore, some patients initially considered ideal for resection are postoperatively confirmed to have advanced-stage disease, resulting in poor prognoses [43]. The assessment of resectability solely involves a morphological evaluation of the extent of vascular involvement by tumours, failing to identify patients with occult metastases and aggressive biology [44, 45]. For these patients, surgical resection entails uncertain benefits. Therefore, ensuring precise preoperative staging of patients is essential for clinicians, complementing resectability assessments and guiding clinical decisions. For patients with early-stage disease who are deemed, suitable for surgical resection, it suggests the necessity for prompt surgical intervention. However, for patients with advanced-stage disease who are still considered candidates for surgical resection, it underscores the importance of active participation in MDT to carefully deliberate the decision for surgical intervention, given the typically poor survival outcomes observed post-surgery in these patients. Therefore, a preoperative staging model for pancreatic cancer was developed based on relatively objective and readily accessible indicators. The objective was to assist clinicians in accurately determining tumour progression stages and effectively predicting prognosis.

There were some limitations to our study. Firstly, the sample size was relatively small, warranting further studies with a larger cohort of patients to validate our findings. Secondly, this study was conducted at a single centre without external or independent validation. Future research initiatives will involve collaborative efforts across multiple centres to augment the model’s stability and robustness. Thirdly, manual tumour segmentation proved time-consuming, highlighting the need to develop automatic segmentation methods in future studies.

## Conclusions

A nomogram approach was developed and validated based on spectral CT imaging. This approach integrates radiomics features with CT spectral parameters (NID-PVP) and clinical factors (tumour diameter), assisting clinicians in preoperatively predicting the PDAC stage. This approach represents a non-invasive, efficient, and feasible tool for PDAC staging, assisting in clinical decision-making for patients with PDAC.

### Electronic supplementary material

Below is the link to the electronic supplementary material.

Supplementary Material 1

Supplementary Material 2

## Data Availability

The datasets used or analyzed during the current study are available from the corresponding author upon reasonable request.

## Abbreviations

AIC: Akaike information criterion
AUC: area under the curve
CA 12 - 5: carbohydrate antigen 12 - 5
CA19-9: carbohydrate antigen19-9
CEA: carcinoembryonic antigen
DCA: decision curve analysis
DLCT: dual-layer spectral detector CT
ICCs: interclass correlation coefficients
ID: iodine density
K- slope: slope of the attenuation curve
LASSO: Least absolute shrinkage and selection operator
mRMR: maximum relevance minimum redundancy
NID: normalized iodine density
PEIs: polyenergetic images
PDAC: pancreatic ductal adenocarcinoma
ROI: region of interest
ROC: receiver operating characteristic curve
Radscore: radiomics score
VMIs: virtual monoenergetic images
Z_eff_: effective atomic numbers
SBI: spectral-based imaging
VIF: variance inflation factor
