# Supplementary material for: A clinical-radiomics nomogram based on dual-layer spectral detector CT to predict cancer stage in pancreatic ductal adenocarcinoma
Source: Cancer Imaging. 2024 May 9;24:55. doi: 10.1186/s40644-024-00700-z (PMC11080083; doi:10.1186/s40644-024-00700-z)
Supplement: Supplementary file 1 — Supplementary Material 1 [file 40644_2024_700_MOESM1_ESM.docx]

**Supplementary Material 1**

**DLCT scan parameters**

All patients underwent pancreatic-protocol CT examination on a DLCT scanner (IQon Spectral CT, Philips Healthcare) with the following parameters: tube voltage, 120 kVp; tube current, modulated with automated exposure control; helical pitch, 0.798; rotation time, 0.5 seconds; collimation, 64 × 0.625 mm; matrix, 512 × 512; reconstructed slice thicknesses, 1.5 mm; and increments, 1.5 mm. After unenhanced scanning, approximately 65-75 mL of iodohexol (350 mg I/mL, Omnipaque, GE Healthcare) was injected into the antecubital vein at 3.5 mL/s via a pump injector followed by a 40-mL saline bolus. Arterial and Portal venous phases were carried out at 25-35 seconds and 60-70 seconds after injection, respectively.

| Supplementary Table 1. Interobserver Agreement for spectral parameters | | |
| --- | --- | --- |
| ICCs | AP | PVP |
| CT_Hu_ | 0.852 | 0.863 |
| ID | 0.882 | 0.897 |
| NID | 0.913 | 0.921 |
| IDD  K-slope  Z_eff_ | 0.889  0.887  0.903 | 0.902  0.905  0.910 |
| AP, arterial phase; PVP, portal venous phase; ID, iodine density; NID, normalized iodine density; IDD, Iodine density difference (ID-AP－ID-PVP); CT, computed tomography; K-slope, the slope of the attenuation curve; Z_eff_, effective atomic number.  ICC: less than0.50, poor; 0.50–0.74, moderate; 0.75–0.90, good; greater than 0.90, excellent. | | |

| **Supplementary Table 2. Baseline characteristics of the training and test cohorts** | | | |
| --- | --- | --- | --- |
| Characteristics | Training cohort（n=122） | Test cohort  （n=51） | *p* value |
| Age（y） | 61.6 ± 8.6 | 59.6 ± 9.9 | 0.183 |
| Gender |  |  | 0.441 |
| Male | 72 (59.0) | 34 (66.7) |  |
| Female | 50 (41.0) | 17 (33.3) |  |
| BMI | 22.2±3.2 | 22.4±3.3 | 0.593 |
| Smoking | 27 (22.1) | 9(17.6) | 0.648 |
| Diabetes | 26 (21.3) | 6 (11.8) | 0.208 |
| Tumor location |  |  | 0.640 |
| Head and neck | 71 (58.2) | 27 (52.9) |  |
| Body and tail | 51 (41.8) | 24 (47.1) |  |
| Tumor diameter |  |  | 0.423 |
| ≤ 2cm | 12 (9.8) | 2 (3.9) |  |
| > 2-4cm | 60 (49.2) | 26 (51.0) |  |
| > 4cm | 50 (41.0) | 23 (45.1) |  |
| CA19-9 (%) |  |  | 0.292 |
| < 37 U/ml | 23 (18.9) | 14 (27.5) |  |
| ≥ 37 U/ml | 99 (81.1) | 37 (72.5) |  |
| CA12-5 (%) |  |  | 0.066 |
| < 35 U/ml | 66 (54.1) | 36 (70.6) |  |
| ≥ 35 U/ml | 56 (45.9) | 15 (29.4) |  |
| CEA (%) |  |  | 0.603 |
| < 5 ug/L | 60 (49.2) | 28 (54.9) |  |
| ≥ 5 ug/L | 62 (50.8) | 23 (45.1) |  |
| CT_Hu-_AP (Hu) | 84.9 ± 30.8 | 85.3 ± 31.3 | 0.946 |
| CT_Hu_-PVP (Hu) | 117.0 ± 36.3 | 114.4 ± 39.4 | 0.673 |
| ID-AP (mg/ml) | 0.6 ± 0.4 | 0.6 ± 0.3 | 0.893 |
| ID-PVP (mg/ml) | 1.0 ± 0.4 | 1.0 ± 0.4 | 0.586 |
| IDD (mg/ml) | 0.8 ± 0.5 | 0.7 ± 0.5 | 0.275 |
| K-slope-AP | 0.7 ± 0.5 | 0.8 ± 0.5 | 0.651 |
| K-slope-PVP | 1.2 ± 0.5 | 1.2 ± 0.6 | 0.916 |
| Z_eff_-AP | 7.6 ± 0.2 | 7.7 ± 0.2 | 0.975 |
| Ze_ff_-PVP | 7.9 ± 0.2 | 7.8 ± 0.3 | 0.604 |
| Note. Data are n (%) or mean (standard deviation); data in parentheses are percentages.  Abbreviation: AP, arterial phase; PVP, portal venous phase; ID, iodine density; NID, normalized iodine density; IDD, Iodine density difference (ID-AP－ID-PVP); CT, computed tomography; CA 19–9, carbohydrate antigen 19–9; CA 12–5, carbohydrate antigen 12–9; CEA, carcinoembryonic antigen. K-slope, the slope of the attenuation curve; Z_eff_, effective atomic number. | | | |

**
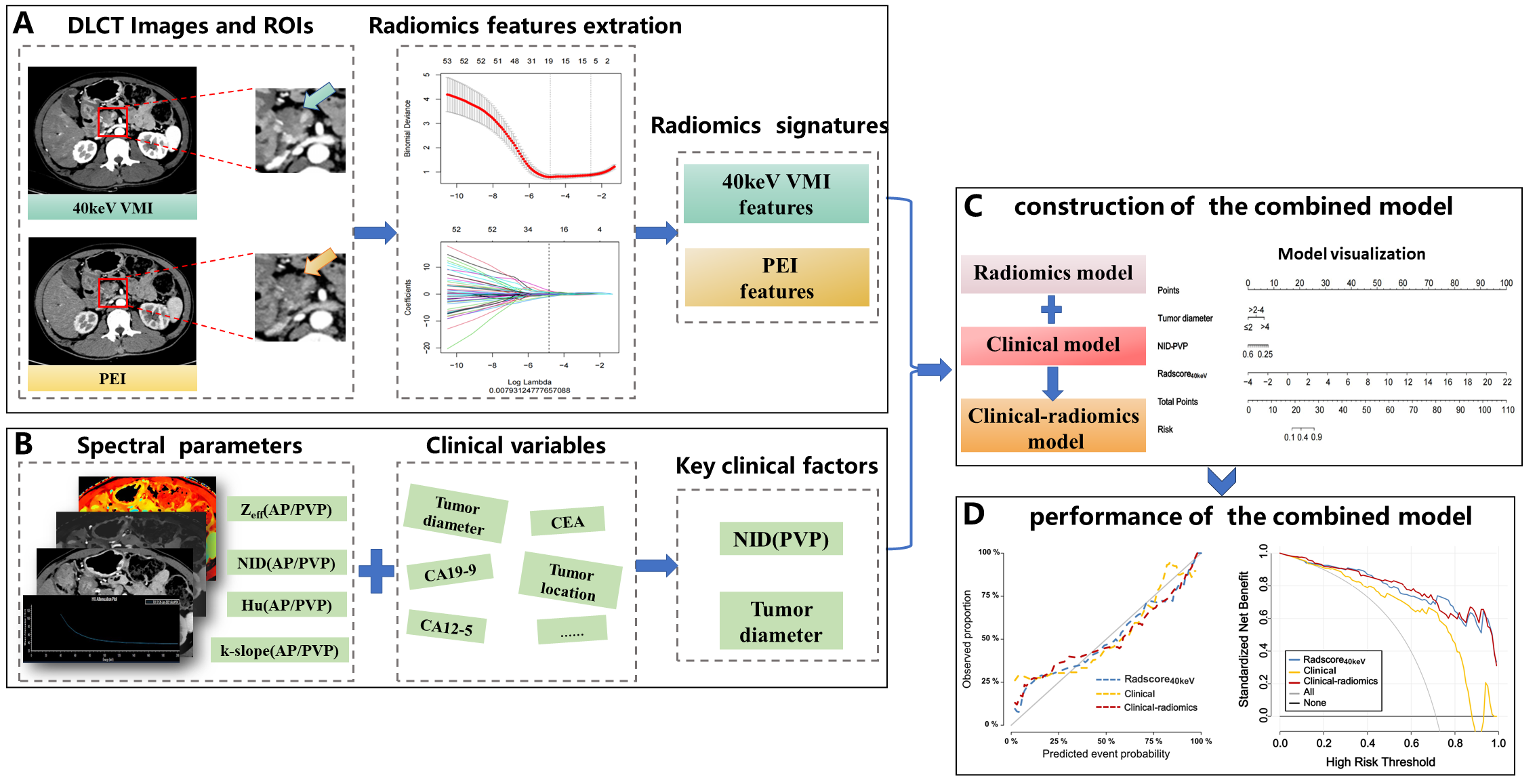
**

**Supplementary Fig. 1.** Workflow of clinical-radiomics nomogram based on DLCT for the stage of patients with pancreatic ductal adenocarcinoma (PDAC). **A.** Radiomics signature construction. **B.** clinical model construction. **C.** construction of the combined model. **D.** performance of the combined model.

**Image preprocessing**

1. ***Image Resampling***: before feature extraction, images were resampled with a voxel size of 1×1×1 mm³ to eliminate interference caused by different devices and scanning parameters on radiomics features.
2. ***Image Discretization***: a method to set a suitable bin width is to extract the feature named first order range such that it remains approximately in this bin range. An absolute discretization was performed with a fixed bin size (bin size = 25).
3. ***Image standardization:***

***
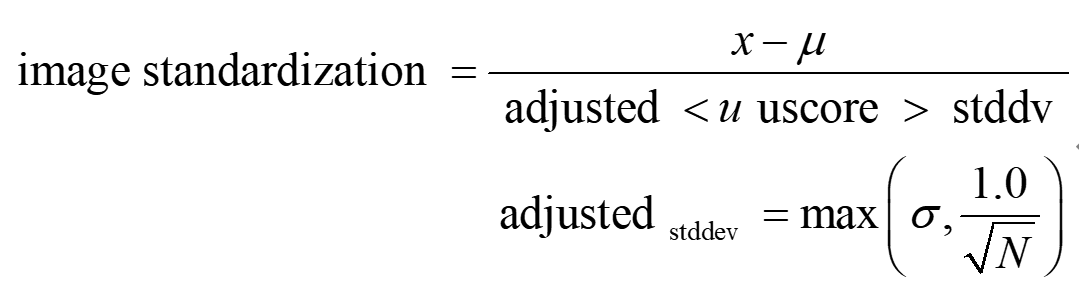
***

1. ***Image normalization:***

***
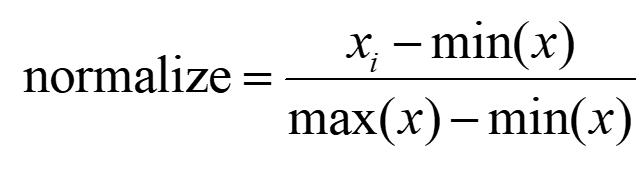
***


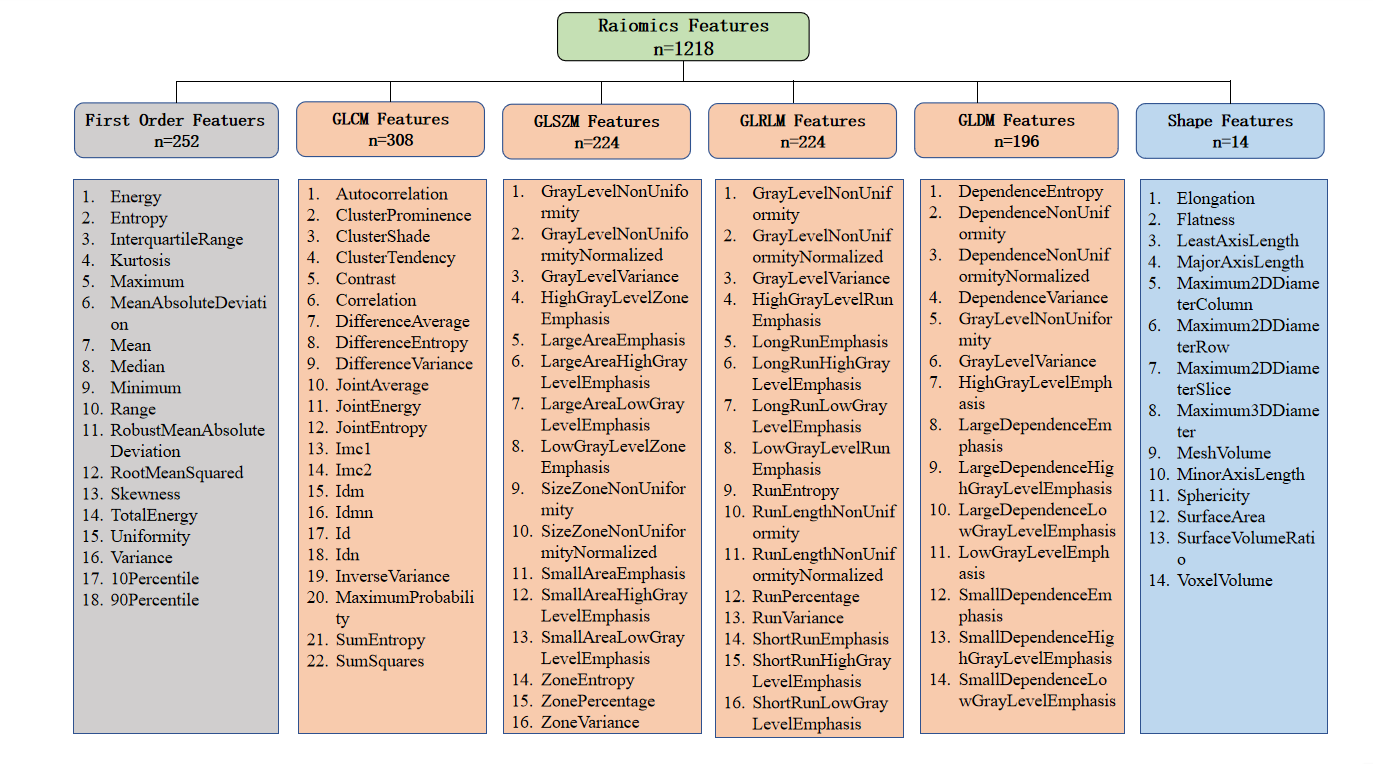
**Supplementary Fig. 2.** 1218 Radiomics features. Including First-order features; GlCM features; GLSZM features; GLRLM features; GLDM features and Shape features.


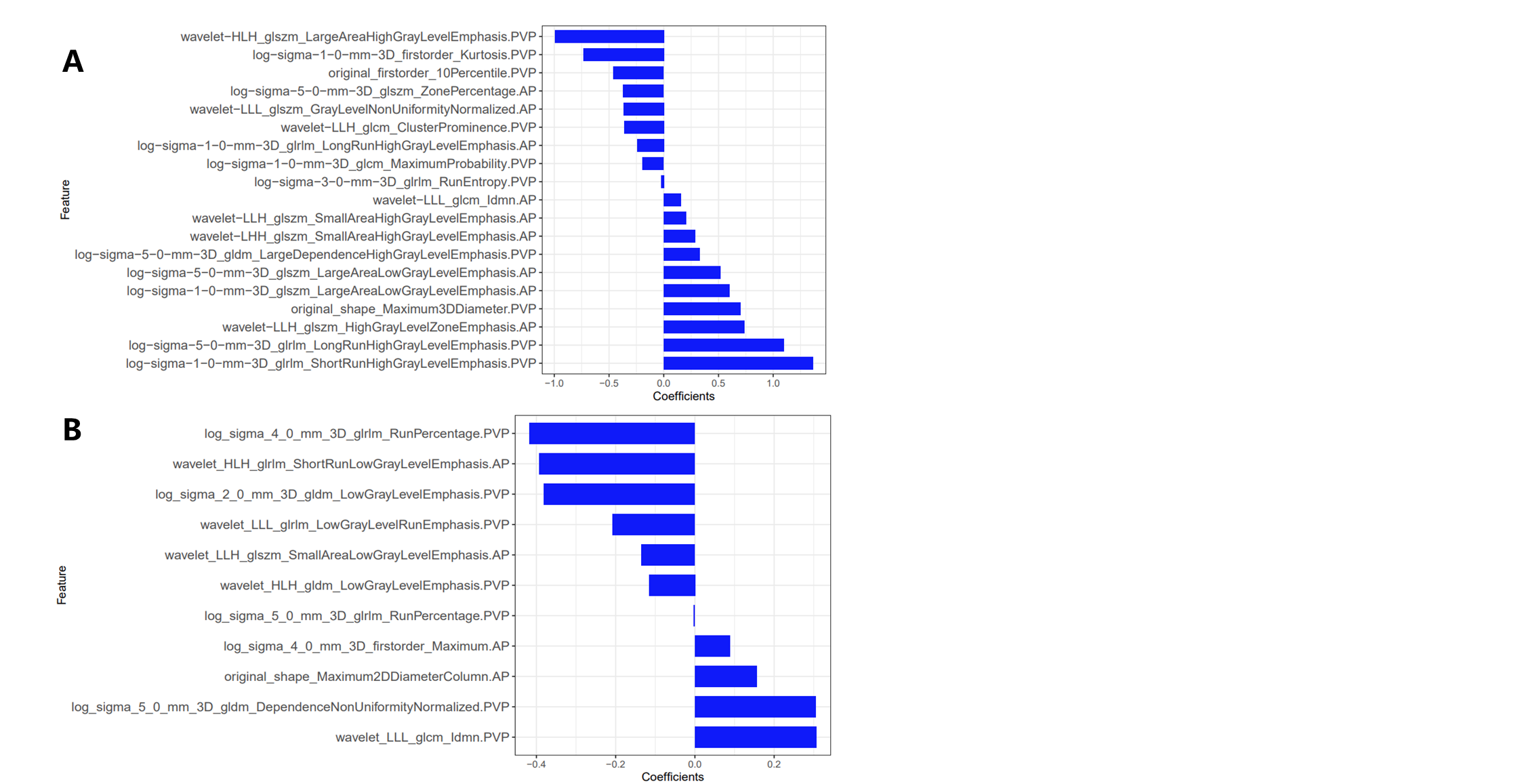


**Supplementary Fig. 3. A.** 40keV VMI-based and **B.**PEI-based radiomics features and their corresponding weight coefficients.


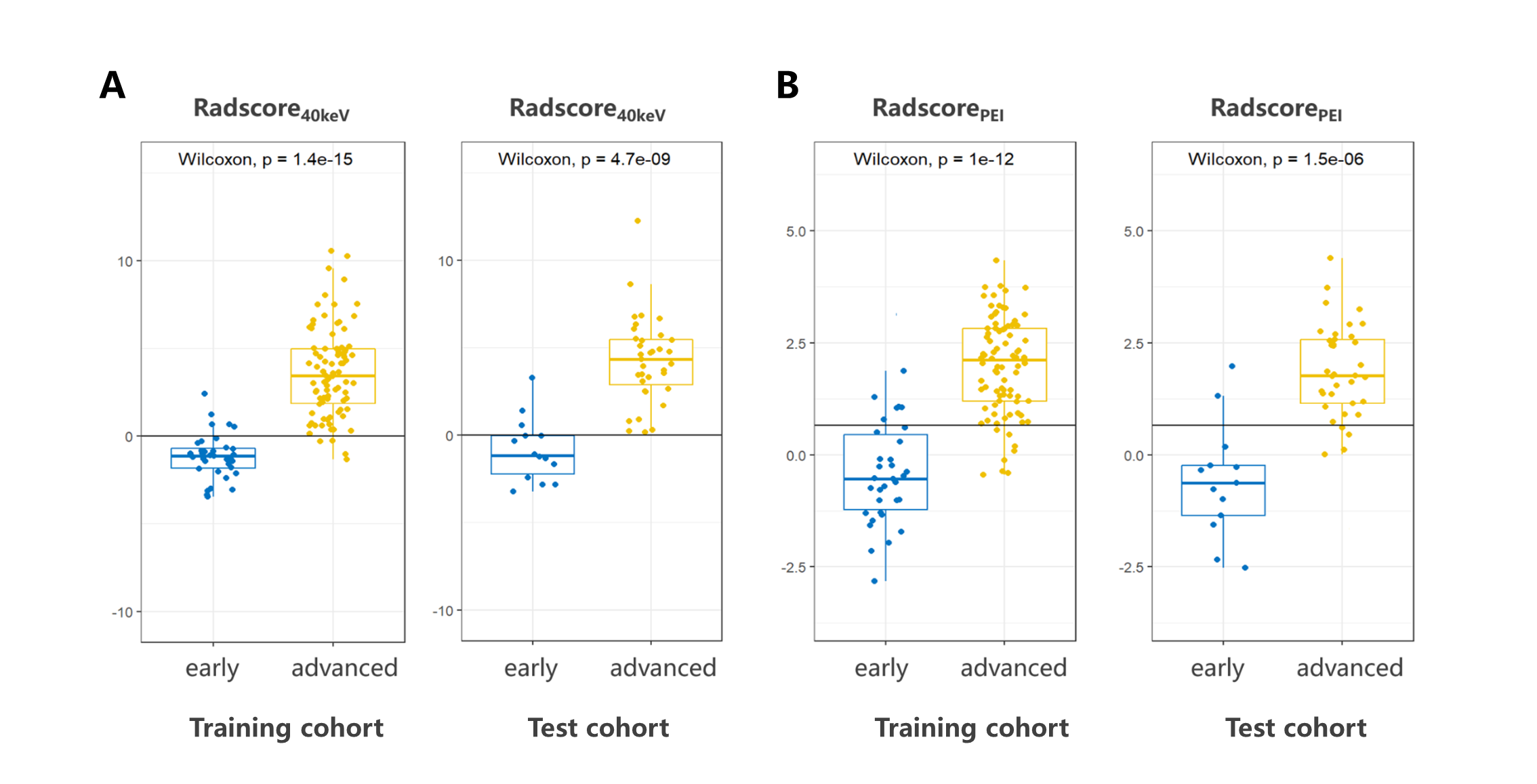


**Supplementary Fig. 4.** **A.** 40keV VMI-based radiomics score (Radscore_40keV_) of PDAC patients in the training and test cohorts. **B.** PEI-based radiomics score (Radscore_40keV_) of PDAC patients in the training and test cohorts. The blue box plot represents the early-stage group. The yellow box plot represents the advanced stage group.


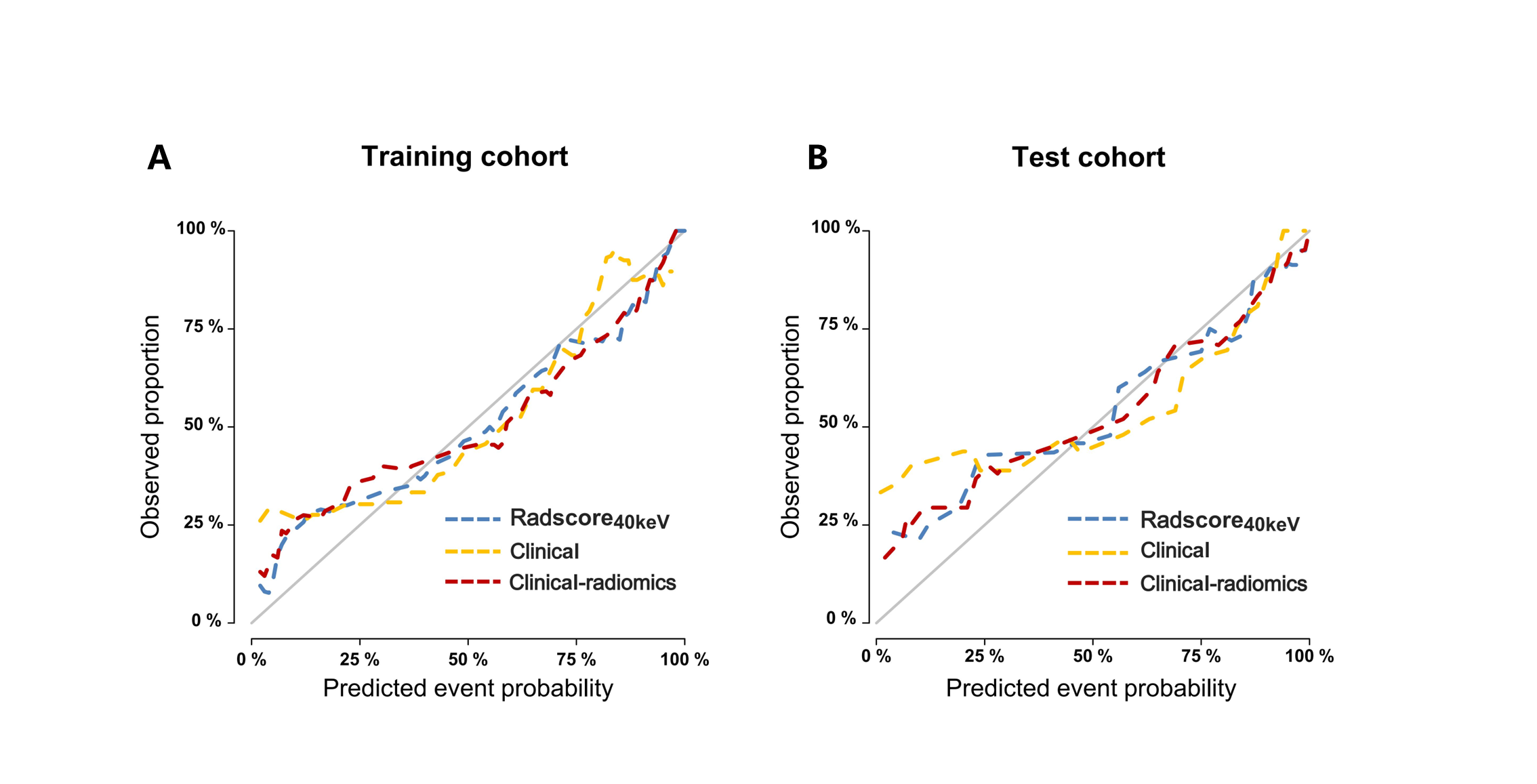


**Supplementary Fig. 5**. Calibration curves for Radscore_40keV_, clinical model and clinical-radiomics model in **A.** the training cohort and **B.** the test cohort. The red line represents the clinical-radiomics model. The blue line represents the Radscore_40keV_ and the yellow line represents the clinical model.
