# Supplementary material for: A clinical-radiomics nomogram based on dual-layer spectral detector CT to predict cancer stage in pancreatic ductal adenocarcinoma
Source: Cancer Imaging. 2024 May 9;24:55. doi: 10.1186/s40644-024-00700-z (PMC11080083; doi:10.1186/s40644-024-00700-z)
Supplement: Supplementary file 2 — Supplementary Material 2 [file 40644_2024_700_MOESM2_ESM.docx]

**Supplementary Material 2**

**The equations of the 40keV VMI-based radiomics models**

*Radscore_40keV_* = 0.702 × original_shape_Maximum3DDiameter. PVP

＋ 0.33 × log-sigma-5-0-mm-3D_gldm_LargeDependenceHighGrayLevelEmphasis. PVP

－ 0.371× log-sigma-5-0-mm-3D_glszm_ZonePercentage. AP

＋ 1.099 × log-sigma-5-0-mm-3D_glrlm_LongRunHighGrayLevelEmphasis. PVP

－ 0.362 × wavelet-LLH_glcm_ClusterProminence. PVP

＋ 1.365 × log-sigma-1-0-mm-3D_glrlm_ShortRunHighGrayLevelEmphasis. PVP

＋ 0.736 × wavelet-LLH_glszm_HighGrayLevelZoneEmphasis. AP

－ 0.994 × wavelet-HLH_glszm_LargeAreaHighGrayLevelEmphasis. PVP

－ 0.734 × log-sigma-1-0-mm-3D_firstorder_Kurtosis. PVP

－ 0.369 × wavelet-LLL_glszm_GrayLevelNonUniformityNormalized. AP

－ 0.245 × log-sigma-1-0-mm-3D_glrlm_LongRunHighGrayLevelEmphasis. AP

－0.194 × log-sigma-1-0-mm-3D_glcm_MaximumProbability. PVP

＋ 0.285 × wavelet-LHH_glszm_SmallAreaHighGrayLevelEmphasis. AP

＋ 0.52 × log-sigma-5-0-mm-3D_glszm_LargeAreaLowGrayLevelEmphasis. AP

－ 0.461 × original_firstorder_10Percentile. PVP

＋ 0.205 × wavelet-LLH_glszm_SmallAreaHighGrayLevelEmphasis. AP

－ 0.025 × log-sigma-3-0-mm-3D_glrlm_RunEntropy. PVP

＋ 0.157 × wavelet-LLL_glcm_Idmn. AP

＋ 0.603 × log-sigma-1-0-mm-3D_glszm_LargeAreaLowGrayLevelEmphasis. AP

＋ 2.398

**The equations of the PEI-based radiomics models**

*Radscore_PEI_* = 0.156 × original_shape_Maximum2DDiameterColumn.AP

＋ 0.307 × wavelet-LLL_glcm_Idmn.PVP

－ 0.135 × wavelet-LLH_glszm_SmallAreaLowGrayLevelEmphasis.AP

＋ 0.305× log-sigma-5-0-mm-3D_gldm_DependenceNonUniformityNormalied.PVP

－ 0.392 × wavelet-HLH_glrlm_ShortRunLowGrayLevelEmphasis.AP

－ 0.002 × log-sigma-5-0-mm-3D_glrlm_RunPercentage.PVP

－ 0.208 × wavelet-LLL_glrlm_LowGrayLevelRunEmphasis.PVP

－ 0.418 × log-sigma-4-0-mm-3D_glrlm_RunPercentage.PVP

＋ 0.088 × log-sigma-4-0-mm-3D_firstorder_Maximum.AP

－ 0.381 × log-sigma-2-0-mm-3D_ gldm_ LowGrayLevelEmphasis.PVP

－ 0.116 × wavelet-HLH_gldm_ LowGrayLevelEmphasis.PVP

＋ 1.278
